# Human sterile alpha motif domain 9, a novel gene identified as down-regulated in aggressive fibromatosis, is absent in the mouse

**DOI:** 10.1186/1471-2164-8-92

**Published:** 2007-04-03

**Authors:** Catherine F Li, Jeffrey R MacDonald, Robert Y Wei, Jocelyn Ray, Kimberly Lau, Christopher Kandel, Rachel Koffman, Sherilyn Bell, Stephen W Scherer, Benjamin A Alman

**Affiliations:** 1 Program in Developmental Biology, the Hospital for Sick Children, 555 University Avenue, Toronto, Ontario, M5G 1X8, Canada; 2 Program in Molecular Genetics and Genomic Biology, the Hospital for Sick Children, 555 University Avenue, Toronto, Ontario, M5G 1X8, Canada; 3 The Department of Surgery, the Hospital for Sick Children, 555 University Avenue, Toronto, Ontario, M5G 1X8, Canada

## Abstract

**Background:**

Neoplasia can be driven by mutations resulting in dysregulation of transcription. In the mesenchymal neoplasm, aggressive fibromatosis, subtractive hybridization identified sterile alpha motif domain 9 (*SAMD9*) as a substantially down regulated gene in neoplasia. *SAMD9 *was recently found to be mutated in normophosphatemic familial tumoral calcinosis. In this study, we studied the gene structure and function of *SAMD9*, and its paralogous gene, *SAMD9L*, and examined these in a variety of species.

**Results:**

*SAMD9 *is located on human chromosome 7q21.2 with a paralogous gene sterile alpha motif domain 9 like (*SAMD9L*) in the head-to-tail orientation. Although both genes are present in a variety of species, the orthologue for SAMD9 is lost in the mouse lineage due to a unique genomic rearrangement. Both SAMD9 and SAMD9L are ubiquitously expressed in human tissues. *SAMD9 *is expressed at a lower level in a variety of neoplasms associated with β-catenin stabilization, such as aggressive fibromatosis, breast, and colon cancers. *SAMD9 *and *SAMD9L *contain an amino-terminal SAM domain, but the remainder of the predicted protein structure does not exhibit substantial homology to other known protein motifs. The putative protein product of *SAMD9 *localizes to the cytoplasm. *In vitro *data shows that *SAMD9 *negatively regulates cell proliferation. Over expression of *SAMD9 *in the colon cancer cell line, SW480, reduces the volume of tumors formed when transplanted into immune-deficient mice.

**Conclusion:**

SAMD9 and SAMD9L are a novel family of genes, which play a role regulating cell proliferation and suppressing the neoplastic phenotype. This is the first report as far as we know about a human gene that exists in rat, but is lost in mouse, due to a mouse specific rearrangement, resulting in the loss of the SAMD9 gene.

## Background

Neoplasia can be driven by a variety of mechanisms. In many cases, oncogenic mutations result in the dysregulation of transcription. This is the case in mutations resulting in β-catenin stabilization in tumors, as stabilized β-catenin activates transcription factors in the Tcf/Lef family. These transcription factors regulate gene expression in a tissue specific manner [1,2]. Aggressive fibromatosis, also known as desmoid tumor, is a locally invasive soft tissue tumor composed of a clonal proliferation of mesenchymal, fibroblast-like, cells [3]. Somatic mutations in either the Adenomatous Polypisis Coli (APC) or β-catenin genes, resulting in the stabilization of β-catenin protein, are present in the majority of lesions. This gives aggressive fibromatosis tumor cells a proliferative advantage through Tcf/Lef dependent transcriptional activation [4,5]. There are a variety of lines of evidences suggesting that differentially expressed genes in aggressive fibromatosis are involved in the development of this tumor. For instance, the differentially expressed genes, *COX-2*, *RHAMM*, *MMP-3*, *TIMP-1 *and *PAI-1 *modulate the size or number of aggressive fibromatosis tumor that form [6-9].

To determine how differentially regulated genes contribute to the neoplastic phenotype in aggressive fibromatosis, suppression subtractive hybridization was used to compare gene expression differences between cell cultures derived from an aggressive fibromatosis tumor with an *APC *mutation and the same cells after transfection of wild-type *APC*. In this comparison, we found that *SAMD9 *was one of the most highly differentially regulated genes, and was upregulated with transfection of the wild-type *APC*. This study focuses on the gene structure and function of *SAMD9 *and its paralogous gene, *SAMD9L*.

## Results

### SAMD9 is differentially regulated in aggressive fibromatosis

Primary cell cultures from an aggressive fibromatosis tumor harbouring a truncating *APC *mutation were established. These were transfected with either a wild-type *APC *gene or a control vector, and successful transfection verified using immunohistochemistry. These cell cultures were established for a previous study to determine the role of the interaction between APC and β-catenin in the regulation of cell proliferation in aggressive fibromatosis. The transfection conditions, mutational analysis, and results of proliferation studies have been previously reported [10,11]. RNA from the cells derived from an aggressive fibromatosis tumor with an *APC *mutation and the same cells after transfection of wild-type *APC *were compared using suppression subtractive hybridization. Both forward (cells transfected with a control vector compared to cells transfected with wild-type *APC*) and reverse (cells transfected with wild-type *APC *compared to cells transfected with a control vector) subtractions were conducted. Clones were identified, and verified using Northern blot. The most differentially expressed clones were sequenced and their identity searched using GenBank.

**Figure 1:**
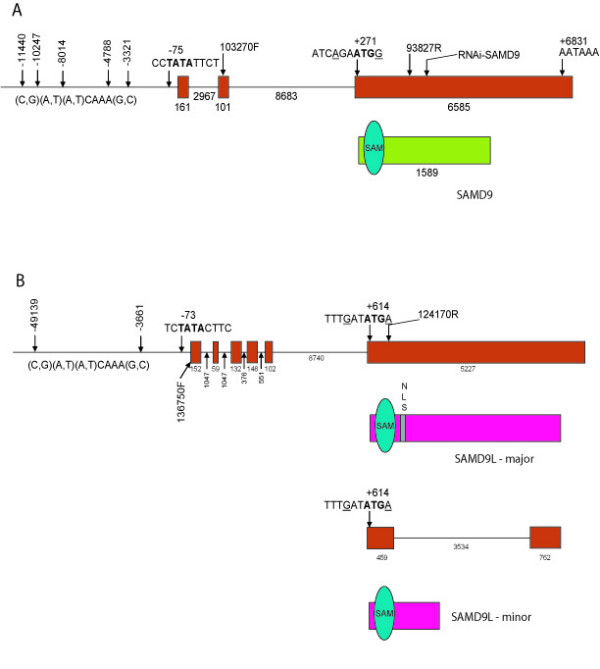
**Gene and protein structure of *SAMD9 *and *SAMD9L***. **A. **Diagram of *SAMD9 *gene structure and predicted protein structure. Exons are shown as red rectangles. The protein is shown in green, with the sterile alpha motif domain as blue oval. **B. **Diagram of *SAMD9L *gene structure and predicted protein structure. Exons are shown in red. The protein is shown as a pink box, with two predicted open reading frames of shown. The sterile alpha motif domain is shown as blue oval. A potential nuclear localization domain is shown as grey box.

In this comparison, the most differentially regulated gene in the reverse subtraction was a novel gene that was initially identified on human chromosome 7 using GenBank sequence data. Both 5'- and 3'- rapid amplification of cDNA ends (RACE) using the sequence of the 200 bp clone isolated from suppression subtractive hybridization, as well as analysis of EST in the region were undertaken to identify the full length of this gene. Subsequent to the start of this work, Strausberg *et al *identified a gene in this region using high through put analysis of EST's and designed the gene sterile alpha motif domain 9 (*SAMD*9) [12]. During this analysis, we also found a nearby gene, which was also subsequently identified by Strausberg *et al *as sterile alpha motif domain 9 like (*SAMD9L*) [12]. Using a search of the DNA sequence database in GenBank, *SAMD9 *and *SAMD9L *were found to match human chromosome 7q21.2. *SAMD9L *is located 5' upstream from the *SAMD9 *gene (Fig. 1). Both genes are coded by the reverse strand with a head to tail orientation.

### Structure of SAMD9 and its paralogue – SAMD9L

Compiling all of the sequence data, we located the most 5'- end sequence for *SAMD9 *in a 5'- RACE clone, and the most 3'- end sequence for *SAMD9 *in one of the EST clones [GenBank: AA628487] [see Additional file 1]. Using primers corresponding to the sequences of the most 5'- and 3'- ends of the *SAMD9 *gene, a predicted 7 kb transcript was amplified from an aggressive fibromatosis tumor cDNA. The full-length of this transcript, the longest transcript that we found, was sequenced [GenBank: AF445355]. The cDNA sequence of *SAMD9 *gene has 6847 bp with a poly-A signal (AATAAA) at 6831 bp downstream from the transcriptional start point (Fig. 1A). Mapping this cDNA sequence to the genomic DNA sequence in the GenBank, the *SAMD9 *gene is 18505 bp in length and contains three exons (161 bp, 101 bp and 6585 bp in length, respectively). There is a TATA signal (CC**TATA**TTCT) at -75 bp and five predicted Lef/Tcf binding elements [(C,G) (A,T) (A,T)CAAA(G,C)] at -11440 bp, -10247 bp, -8014 bp, -4788 bp and -3321 bp. The clones isolated from 5'- and 3'- RACE of the *SAMD9 *gene were sequenced. Comparing the 5'- and 3'- RACE sequences to the genomic DNA sequence from GenBank, different transcription initiation sites and polyadenylation sites of *SAMD9 *are noted, and there are at least two alternative splices.

During the analysis of EST sequences near the initial clone, we identified a second gene,*SAMD9L*, which is located 12740 bp distal to *SAMD9*. Using the sequences from the most 5'- and 3'- EST in the region [Celera Database: THC 511215 and THC 513290, respectively] [see Additional file 1], the full-length sequence of the *SAMD9L *was amplified from an aggressive fibromatosis tumor cDNA, and sequenced [GenBank: AF474973]. The cDNA sequence of *SAMD9L *gene has 5821 bp with a poly-A signal (AATAAA) at 5659 bp downstream from the transcriptional start point (Fig. 1B). Mapping this cDNA sequence to the genomic DNA sequence in GenBank, the *SAMD9L *gene is 17583 bp in length, including six exons (152 bp, 59 bp, 132 bp, 148 bp, 102 bp and 5228 bp in length, respectively). There is a TATA signal (TC**TATA**CTTC) at -73 bp and two predicted Lef/Tcf binding elements [(C,G) (A,T) (A,T)CAAA(G,C)] at -49139 bp and -3661 bp.

There are alternatively spliced transcripts in both *SAMD9 *[GenBank: AF453311] and *SAMD9L *[GenBank: AY195582-195587, DQ068177] [see Additional file 1]. These alternative splices lead to either inclusion or exclusion of an exon due to the usage of a different 5' donor splice site or a different 3' acceptor splice site. This alternative splicing also changes the internal coding region due to an in-frame insertion or deletion in both *SAMD9 *and *SAMD9L*. In the case of *SAMD9*, non-canonical dinucleotides AT and TC are used as donor and acceptor splice sites for intron 3, and there is an in-frame canonical pair of the donor and acceptor (GT and AG) splice sites near by. This alternative splicing changes the internal coding region due to an in-frame deletion, leading to the exclusion of sequence coding for a putative protein structure domain – sterile alpha motif (SAM) domain near the N-terminus.

The putative protein structures of both genes were determined using the open reading frame finder program on the National Center for Biotechnology Information (NCBI) website [13], and an open reading frame was identified in the last exon of both genes. The two genes share 78% homology at the DNA sequence level in this exon. The putative protein sequences were analyzed using the Conserved Domain search program in NCBI website [14] for *SAMD9*, the Motif Scan program from Swiss Institute of Bioinformatics [15-17] and the Predict Protein server from European Molecular Biology Laboratory [18] websites for *SAMD9L*. Both protein products contain a sterile alpha motif (SAM) domain near the N-terminal region. Otherwise, there is no close homology to other well-characterized proteins or protein motifs. Based on a comparison of the SAM domain sequence of *SAMD9 *to the homology modeling of the RNA-binding *Smg *SAM domain [19], the SAM domain of *SAMD9 *lacks the residues that are essential for binding RNA, while it has 98% sequence homology with the Ephrin-B2-receptor SAM domain, which forms homo-oligomerization and provides a platform for the formation of larger protein complexes [20].

### SAMD9 and SAMD9L are ubiquitously expressed

**Figure 2:**
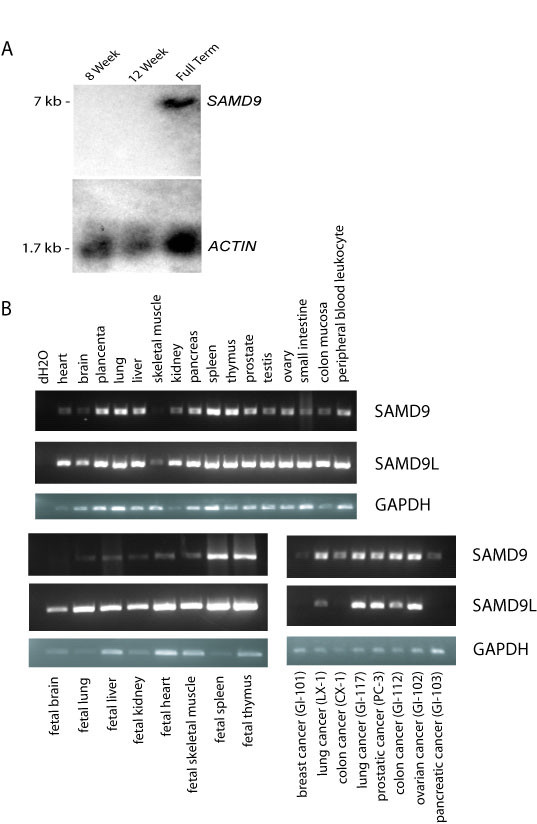
**Expression of *SAMD9 *and *SAMD9L *in multiple tissue types**. **A. **Northern blot from total RNA of 8 weeks, 12 weeks and full term human placenta probed with 3'UTR of *SAMD9*. A 7 kb transcript was detected in full term human placenta. **B. **Human adult, fetal, and tumor multiple tissue cDNA panels were amplified with the sequences at 3'UTR of *SAMD9*, *SAMD9L *or *GAPDH *as primers. *SAMD9 *was expressed in all human adult, fetal and tumor tissues that were available from human multiple tissue cDNA panels except for fetal brain and breast cancer. It was expressed at a very low level in skeletal muscle and pancreatic cancer. *SAMD9L *is expressed in all human adult, fetal and tumor tissues that were available from human multiple tissue cDNA panels, except breast cancer, colon cancer, and pancreatic cancer.

The expected 7 kb transcript of *SAMD9 *was identified by Northern analysis using RNA from different age placentas and a 1729 bp PCR amplified probe encompassing the 3'UTR (Fig. 2A). The expression pattern of *SAMD9 *in multiple tissues was investigated using a human multiple tissue cDNA panel (Clontech, USA) and RT-PCR. The primers were designed to recognize sequences at 3'UTR of *SAMD9*. Expression was detected in all human adult, fetal and tumor tissues that were tested, except for fetal brain. Only a very low signal was detected in skeletal muscle (Fig. 2B). Using the sequences at 3'UTR of *SAMD9L *as primers, *SAMD9L *was also found to be expressed in all tissues, except for the tumor types, colon cancer (CX-1), breast cancer (GI-101) and pancreatic cancer (GI-103) (Fig. 2B).

### SAMD9 and SAMD9L are expressed at lower levels in tumors

**Figure 3:**
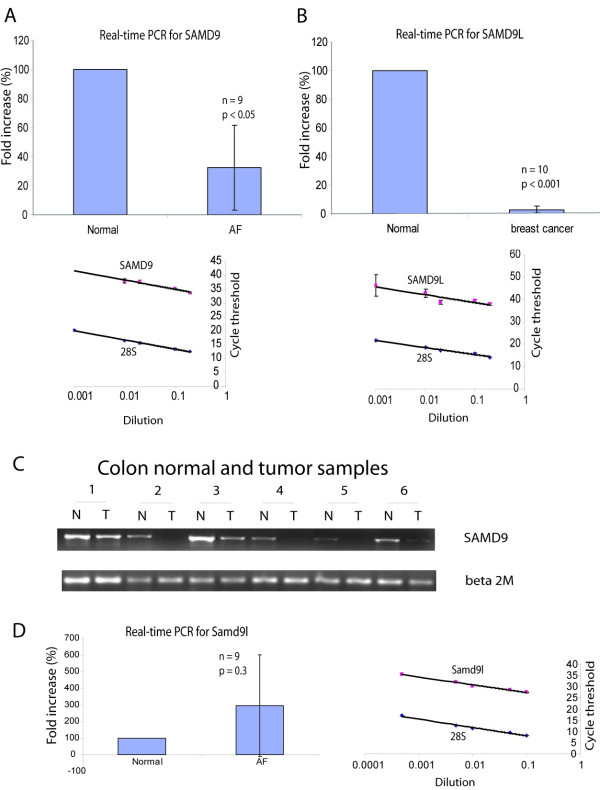
**Lower level of expression of *SAMD9 *and *SAMD9L *in neoplasia**. **A. **Validation curves for the primer sets of *SAMD9 *and 28S rRNA, and real time quantitative PCR of *SAMD9*. *SAMD9 *and 28S rRNA have similar amplification efficiency since the slope was very similar for both curves. *SAMD9 *was expressed at a lower level in aggressive fibromatosis (AF) tumors compared to normal fibrous tissues from same patients. **B. **Validation curves for the primer sets of *SAMD9L *and 28S rRNA, and real time quantitative PCR of *SAMD9L*. *SAMD9L *and 28S rRNA have similar amplification efficiency since the slope was very similar for both curves. *SAMD9L *was expressed at a lower level in breast cancers compared to normal breast epithelial tissues from same patients.**C. **Semi-quantitative RT-PCR of *SAMD9*. *SAMD9 *was under-expressed in some cases of colon cancer compared to the normal colon mucosa from same patients. Real time PCR data showed no significant difference in expression level between breast and colon cancer and normal controls as a group. **D. **There was no significant difference in expression of *SAMD9L *between aggressive fibromatosis samples and normal controls tissues.

To determine the level of expression of *SAMD9 *in neoplasia, semi-quantitative RT-PCR and real-time quantitative PCR were performed using the sequence at 5'UTR of *SAMD9 *as a sense primer and a sequence at the beginning of the open reading frame of *SAMD9 *as an anti-sense primer. Expression was compared between tumor and normal control tissues in cases of aggressive fibromatosis, breast cancer and colon cancer. *SAMD9 *was expressed at a lower level (about 33% the level for normal control tissues) in aggressive fibromatosis (32.22% ± 29.18% vs 100%, p < 0.05 for real-time PCR, Fig. 3A). Expression of *SAMD9 *was lower in 20% cases of breast cancer and 35% cases of colon cancer than in the normal control tissues (Fig. 3C). In three cases of colon cancer, there was no RT-PCR product detected. However, there was no significant difference in expression between colon cancers and normal control tissues or breast cancers and normal control tissues when analysed as a group using real-time PCR. Expression studies of *SAMD9L *were also conducted using a sequence at 5'UTR which is common for all of the splice variants except one, as a sense primer and sequence at the beginning of the open reading frame as an anti-sense primer. There was no statistically significant difference in *SAMD9L *expression between aggressive fibromatosis or colon cancer and normal control tissues. However, the expression of *SAMD9L *was lower in breast cancers than in healthy breast epithelial tissues from the same patients (2.81% ± 2.40% vs 100%, n = 10, p < 0.001, Fig. 3B). There was no significant difference in expression of *SAMD9L *between aggressive fibromatosis samples and normal controls tissues (Fig. 3D). While two genes are expressed at a lower level in tumors, there is a discrepancy between the levels of expression of the two genes in different tumor types. For instance, *SAMD9 *is expressed at lower levels in aggressive fibromatosis, while *SAMD9L *is not.

### SAMD9 is a cytoplasmic protein

**Figure 4:**
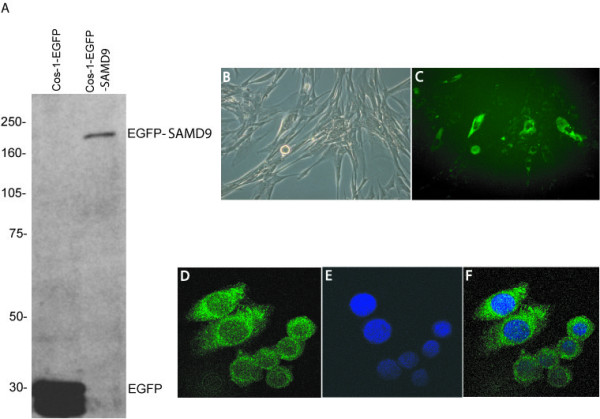
***SAMD9 *protein is expressed and localized to the cytoplasm**. **A. **Western analysis for the SAMD9-EGFP fusion protein. The *SAMD9 *fusion protein was detected as a predicted 200 kDa band in Cos-1 cells, using an antibody to GFP. **B, C. **Expression of *SAMD9 *putative protein in MRC-5 cells. N-terminal EGFP tagged *SAMD9 *was detected in the cytoplasm of MRC-5 cells when transiently transfected. **B. **Light field view. **C. **Fluorescent microscopy view for FITC. **D, E, F. **Expression of *SAMD9 *putative protein in SW480 cells. N-terminal EGFP tagged *SAMD9 *localized to the cytoplasm of SW480 cells. **D. **Fusion protein detected under fluorescent wave length for FITC. **E. **Cell nucleus detected under fluorescent wave length for DAPI. **F. **Merged view of image D and E.

In order to investigate the cellular localization of the putative *SAMD9 *protein, the open reading frame of the *SAMD9 *gene was cloned, an N-terminal EGFP tag was added and subcloned into a CMV driven vector pLP-EGFP-C1 (Clontech, USA). This vector was transfected into Cos-1 cells, and using an antibody to EGFP, the fusion protein was detected as a 200 kDa band (Fig. 4A). To determine its localization in a variety of cell types, it was transfected into a human fetal fibroblast cell line, MRC-5, and a colon cancer cell line, SW480. The EGFP fusion protein was detected diffusely in the cell cytoplasm (Fig. 4B–F).

### RNAi for SAMD9 can be used to down regulate its expression

RNA interference was utilized to study the cellular function of *SAMD9*. RNA interference was designed by searching the public available sequence database to find a 19 bp oligonucleotide sequence specific for *SAMD9 *and inserting into a pSUPER-RNAi-EGFP expression vector to generate the *SAMD9 *RNA interference vector (pSUPER-RNAi-SAMD9). One of the sequences is 846 bp after the start of translation, and other is 2371 bp after the start of translation. The pSUPER-RNAi-EGFP expression vector was generated based on pEGFP-C1 (Clontech, USA) and pSUPER [21]. A nucleotide substitution from G to A at the 9^th ^position of this 19 bp oligonucleotide results in abolishing of its specificity to *SAMD9 *and was used as negative control after subcloned into a pSUPER-RNAi-EGFP expression vector to generate the negative control vector (pSUPER-RNAi-SAMD9-mut9).

**Figure 5:**
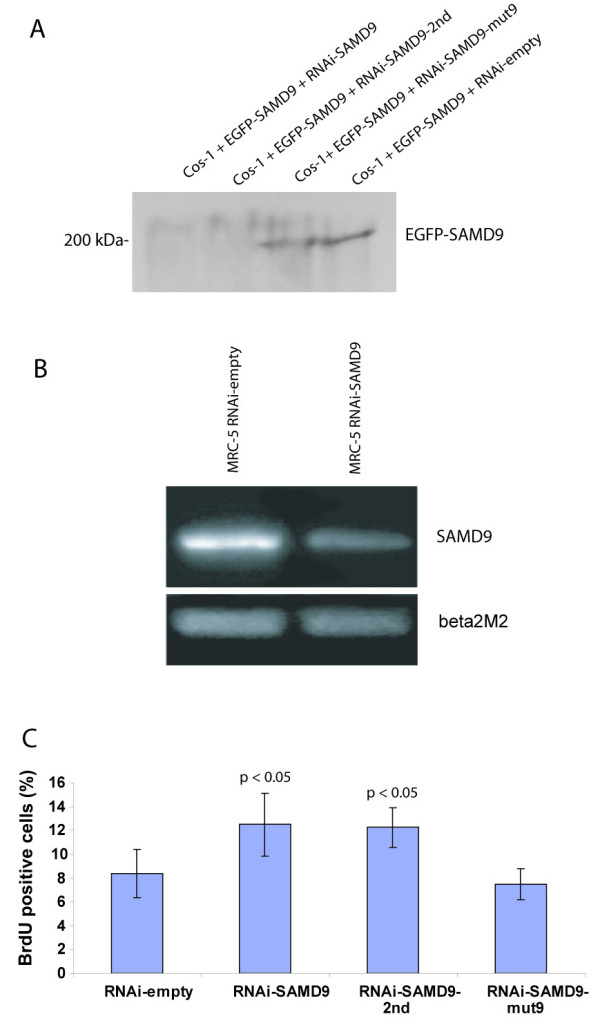
***SAMD9 *regulates cell proliferation**. **A. **RNAi for *SAMD9 *is effective in reducing protein expression. A 200 kDa band for the *SAMD9 *fusion protein was detected in Cos-1 cells after co-transfection of pLP-EGFP-SAMD9 with either pSUPER-RNAi-SAMD9-mut9 or pSUPER-RNAi-EGFP, but it is not detected after co-transfection with either pSUPER-RNAi-SAMD9 or pSUPER-RNAi-SAMD9-2^nd^. The expression of the EGFP-SAMD9 fusion protein was reduced in Cos-1 cells with RNA interference of *SAMD9*. **B. **Semi-quantitative RT-PCR for *SAMD9 *expression in MRC-5 cells. *SAMD9 *expression was reduced after RNA interference. **C. **Proliferation rate (Brdu incorporation percent) increased after RNA interference of *SAMD9 *in MRC-5 cells.

*SAMD9 *expression reduced by RNA interference vector for *SAMD9 *was tested by co-transfection of an N-terminal EGFP tagged *SAMD9 *expression vector with either one of the two *SAMD9 *RNA interference vectors, RNA interference vector with mutated *SAMD9 *sequence to null its function as negative control, or its control empty vector into Cos-1 cells. The reduction of *SAMD9 *expression was confirmed using Western blot with anti-GFP (Santa Cruz, USA) for the fusion putative *SAMD9 *protein. A 90% reduction in expression of *SAMD9 *fusion protein by RNA interference was detected as measured using densitometry (Fig. 5A). The RNA interference vector that express EGFP was also expressed in SW480 cells along with an N-terminal HA tagged *SAMD9 *expression vector or its control vector. A 90% reduction in expression of *SAMD9 *fusion protein by RNA interference was detected (data not shown). Activity of the RNA interference vector for *SAMD9 *was further verified by the reduction of *SAMD9 *RNA expression in MRC-5 cells (Fig. 5B).

### SAMD9 regulates cell proliferation and apoptosis

Because β-catenin stabilization is known to regulate fibroblast proliferation, motility and invasiveness [11], we studied the effect of down-regulating *SAMD9 *on these parameters. Proliferation rate, as measured by BrdU incorporation, was increased after RNA interference of *SAMD9 *in MRC-5 cells (12.5% ± 2.66% with the 1^st ^*SAMD9 *RNA interference vector, 12.2% ± 1.63% with the 2^nd ^*SAMD9 *RNA interference vector, and 7.5% ± 1.29% with the control vector, p < 0.01, Fig. 5C). There was not a statistical difference in cell motility as measured by the number of cells passing through the membrane of the culture insert (1.7 ± 0.65 or 2.3 ± 1.31 with RNA interference of *SAMD9 *vector vs 0.67 ± 0.65 with control vector). Cell invasion was increased, as measured by the number of cells passing through the membrane of the Matrigel invasion chambers, after *SAMD9 *expression was decreased in MRC-5 cells by RNA interference (16 ± 5.19 with RNA interference of *SAMD9 *vector vs 5.7 ± 1.73 with control vector, p < 0.05). These studies were also undertaken in Cos-1 cells, and showed a similar reduction in proliferation rate after over-expression of the putative *SAMD9 *protein in Cos-1 cells (20.7% ± 21.9% vs 76.8% ± 20.2%, p < 0.005). Taken together, this shows that *SAMD9 *plays a role regulating cell proliferation.

**Figure 6:**
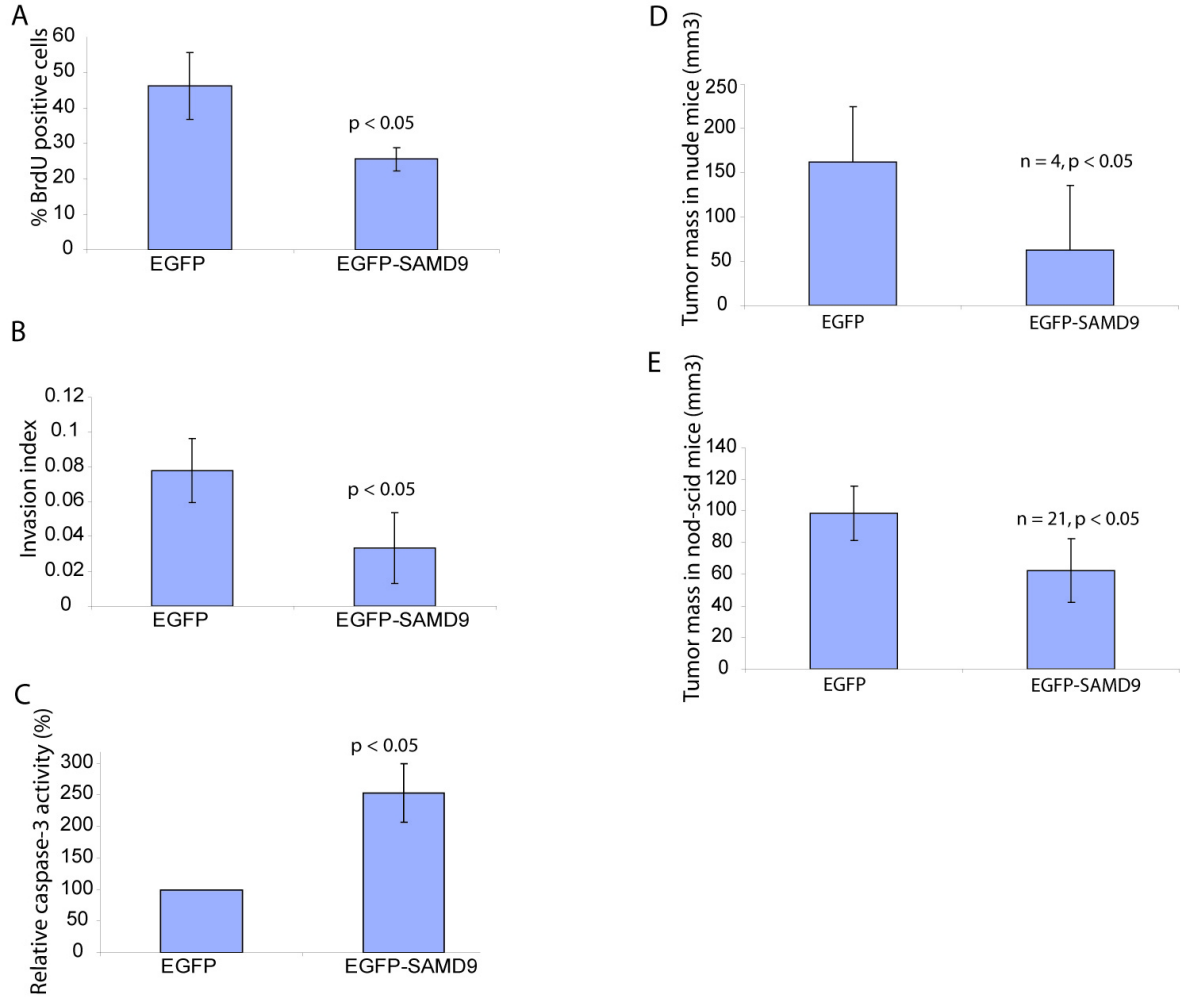
***SAMD9 *reduces tumor size**. **A. **Increased expression of *SAMD9 *reduced SW480 cell proliferation rate as detected by Brdu incorporation percent. **B. **Increased expression of *SAMD9 *reduced the invasion index of SW480 cells. **C. **Increased expression of *SAMD9 *increased caspase activity in SW480 cells. **D and E. **The tumor volume formed from SW480 xenografts was reduced with increased expression of *SAMD9 *when transplanted into nude mice (**D**) or Nod-scid mice (**E**).

To explore the effect in neoplasia, cells from the SW480 colon cancer cell line were transfected with a *SAMD9 *expression vector to determine if increasing expression of this gene would suppress the neoplastic behavior in these cells. Upon transfection of the construct, cell proliferation was reduced (25.6% ± 3.3% vs 46.2% ± 9.4%, p < 0.01, Fig. 6A). Cell invasion index calculated as the ratio of the total number of nuclei counted in the membrane for invasion assay to that in the membrane for motility assay was reduced (0.0335 ± 0.0203 vs 0.0779 ± 0.0183, p < 0.05, Fig. 6B), while cell apoptosis, as indirectly measured by relative caspase-3 activity was increased (253.11% ± 45.90% of baseline, p < 0.005, Fig. 6C). The relative caspase-3 activity was also reduced in Cos-1 cells after expression of the *SAMD9 *siRNA construct (53.76% ± 0.99% of the baseline, p < 0.01). Thus expressing *SAMD9 *at higher levels causes cell effects which would be expected to suppress the neoplastic phenotype.

### SAMD9 regulates tumor growth

To investigate the role of *SAMD9 *in tumor growth, the colon cancer cell line, SW480, was transfected with *SAMD9 *expression vector or its control empty vector. The stably transfected SW480 cells were injected into the immune deficient mice. The tumor volume of the xenograft from SW480 cells over-expressing *SAMD9 *was reduced in both nude mice (62.5 ± 73.5 mm^3 ^vs 162.5 ± 61.7 mm^3^, n = 4, p < 0.05, Fig. 6D) and NOD-SCID mice (62.4 ± 20.3 mm^3 ^vs 98.6 ± 17.1 mm^3^, n = 21, p < 0.01, Fig. 6E). To verify the role of *SAMD9 *on neoplasia, we performed experiments when the expression of *SAMD9 *was lowered using RNA interference. The tumor volume of the xenograft from SW480 cells expressing the RNA interference of *SAMD9 *was increased in nude mice (310 ± 19.6 mm^3 ^vs 270 ± 24 mm^3^, n = 5, p < 0.05).

### Orthologous genes of SAMD9 and SAMD9L

The orthologous genes were identified for *SAMD9 *and *SAMD9L *by searching the NR and swissprot databases for best reciprocal matches using human *SAMD9 *and *SAMD9L *protein sequences. Genome sequences for all species available were searched by BLAT at the University of California at Santa Cruz genome browser [22]. The results were summarized in Table 1 and the phylogram [see Additional File 1] was constructed using multiple sequence alignment of orthologous genes identified with ClustalW program from European Bioinformatics Institute website [23]. The default parameters from website documentation were used [24]. Based on their sequence similarity, *SAMD9 *and *SAMD9L *appear to have originated from a common ancestor by an ancient gene duplication. Since there are orthologous genes of both *SAMD9 *and *SAMD9L *in chimpanzees, dogs and rats, but not in chicken, frog or fish species, this duplication event likely occurred after the mammalian radiation. The genomic structures of both *SAMD9 *and *SAMD9L*, including the order and orientation of genes within the genomic region and the relative size of the intergenic region, are conserved in all available mammalian genome sequences including opossum, indicating the gene duplication event occurred between 175 to 200 MYA [25]. No evidence for either gene was found in lower eukaryotes such as Drosophila, C. elegans or yeast.

### SAMD9 is lost in the mouse lineage

**Figure 7:**
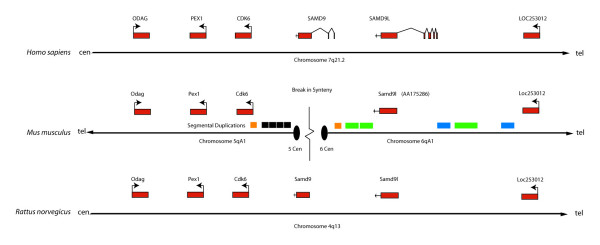
**Diagram for the genomic region encompassing the loci of *SAMD9 *and *SAMD9L *in human, mouse and rat along with the segmental duplications**. Exons are shown as a red box. Segmental duplications are shown as orange, green, blue and grey boxes. Two orange segmental duplications may mediate chromosomal breakage with subsequent deletion of *SAMD9*, and the centromeres of chromosome 6 and 5 are formed at the sites of breakage. The grey segmental duplications, sharing the sequence among numerous centromeric loci, are accumulated at the centromere of chromosome 5. The green and blue segmental duplications flanking the *SAMD9L *are mouse specific based on sequence similarity, while the orange and grey segments have occurred at about the same time as the divergence of mouse and rat.

There were several overlapping ESTs matching the open reading frame for *SAMD9L*, and no ESTs corresponding to the open reading frame for *SAMD9 *in the mouse genome. This was confirmed by bioinformatic searches of all genomic sequences and partial genome sequences (EST libraries). The 168 kb region in human chromosome 7, in which *SAMD9 *is located, has no match in the mouse genome. A syntenic map of the region was constructed for human, mouse and rat. A break of synteny at the *SAMD9 *locus was determined in mouse, with distal genes mapping to mouse chromosome 6, and the proximal genes mapping to mouse chromosome 5. The mouse specific rearrangement resulted in new centromere formation at the site of breakage and the subsequent loss of the *SAMD9 *gene. The *SAMD9L *region contains numerous gaps in the mouse genome assembly, and an enrichment of mouse specific segmental duplications (Fig. 7, also see Additional file 1). The relatively poor draft quality of the rat genome does not allow for high resolution mapping of this locus, but there is evidence to support the existence of both *SAMD9 *and *SAMD9L *genes in rats with no large genomic rearrangements, segmental duplications or breaks in synteny although numerous gaps do exist (Fig. 7, also see Additional file 1). There are no gaps or segmental duplications at this locus in the human genome, determined by searching the Human Genome Segmental Duplication Database.

## Discussion

Here we identified a novel gene *SAMD9 *and its paralogue *SAMD9L *located in human chromosome 7q21.2. These genes are expressed ubiquitously in human tissues. *SAMD9 *is expressed at a lower level in aggressive fibromatosis and some cases of breast and colon cancer, while *SAMD9L *is expressed at a lower level in breast cancer, compared to normal control tissues from the same patients. The putative protein of *SAMD9 *is localized in the cytoplasm. Our *in vitro *data suggests *SAMD9 *regulates cell proliferation rate in both normal and tumor cell lines. Over-expression of *SAMD9 *in SW480 cells reduced the volume of tumors that formed in nude mice and NOD-SCID mice, while the decreased expression in SW480 cells increased the tumor volume formed in nude mice suggesting a role suppressing the neoplastic phenotype.

Very recently, it was found that *SAMD9 *is mutated in the inherited condition, normophosphatemic familial tumoral calcinosis [26]. In the work of Topaz *et al*, there is a likely inadvertent error, as when comparing the protein sequences in the publication with the swissProt/NCBI database, it was found that the mouse *SAMD9 *sequence reported is actually a predicted rat *SAMD9 *protein sequence [GenBank: XP_575365] and the rat *SAMD9 *sequence reported is the rat predicted *SAMD9L *protein sequence [GenBank: XP_001069386]. While conclusions in their manuscript on the species conservation of *SAMD9 *are incorrect, the occurrence of soft tissue lesions in this inherited condition supports our notion that *SAMD9 *plays an important role in cell processes that are important in neoplasia.

There are alternatively spliced transcripts in both *SAMD9 *and *SAMD9L*. The mechanism for alternative splice of *SAMD9 *is unusual in that it uses the non-canonical dinucleotides AT and TC as donor and acceptor splice sites for intron 3, and there is an in-frame canonical pair of the donor and acceptor (GT and AG) splice sites near by. Although only the canonical GT-AG, and the non-canonical GC-AG and AT-AC pairs can recruit the splicing machinery effectively, Burset *et al *suggested that the other non-canonical pairs can function exclusively in association with a canonical pair, which shares its properties with the neighbour, as some kind of parasitic splice sites [27]. This alternative splice results in the in-frame deletion of the sequence coding for the sterile alpha motif (SAM) domain near the N-terminus of *SAMD9*. Although it is only detected in a quarter of the normal and tumor tissues tested, it has potential to play a dominate-negative role in the functioning of the main common *SAMD9 *transcript with the SAM domain. The alternative splicing increases the functional complexity of this novel gene, but the function significance of these alternative splicing variants needs further investigation.

While we did not investigate the regulation of expression in *SAMD9 *and *SAMD9L*, there are Lef/Tcf binding elements predicted in the genomic sequence upstream of the transcriptional start point for both genes. We also found that the expression of these genes is disregulated in neoplasms driven by β-catenin mediated signaling. Although this suggests *SAMD9 *may be regulated by β-catenin, formal promoter studies are needed to verify this possibility.

There is an N-terminal sterile alpha motif (SAM) domain in the predicted putative proteins of *SAMD9 *and *SAMD9L*. The SAM domain sequence of *SAMD9 *was compared using the homology modeling of the RNA-binding *Smg *SAM domain [19]. This showed that the SAM domain lacks the residues that are essential for binding RNA, while it has 98% sequence homology with the Ephrin-B2-receptor SAM domain, which is important in homo-oligomerization and acts as a platform for the formation of larger protein complexes [20]. This raises the possible that *SAMD9 *may have a similar function participating in protein complexes.

Human chromosome 7q21.2 is located at an evolutionary breakpoint between mouse chromosome 5 and 6, which was localized after human-mouse comparative mapping of the genomic region containing *CDK6*, i.e., human genes' centromeric to *CDK6 *map to proximal mouse chromosome 5, while those telomeric to the open reading frame for *SAMD9L*, map to proximal mouse chromosome 6 [28]. Based on our bioinformatics analysis, there are chimpanzee, dog, and rat orthologous genes for both *SAMD9 *and *SAMD9L*. Although there is a mouse ortholog of *SAMD9L*, *SAMD9 *is lost in the mouse genome. This is the first report as far as we know about a human gene that exists in rat, but is lost in mouse.

Four human genes, collagen XXI (*COL21A1*), serine-threonine kinase family member (*STK17A*), G-protein coupled receptor family member (*GPR145*) and ras homologue gene family member I (*ARH I*), located in regions corresponding to evolutionary breakpoints in rodents are absent in both mouse and rat genomes due to an unexpected consequence of evolutionary chromosome rearrangement at the evolutionary breakpoint and lost during evolution [29]. *SAMD9 *locates in a 168 kb region on human chromosome 7, which has no match in the mouse genome, and at the break in synteny in the mouse genome. But there is no break in synteny at this locus in the rat genome. Mouse-human breakpoint absent in the human-rat comparison suggests the rearrangement is specific to the mouse lineage, i.e. mouse-specific breakpoint. There are no segmental duplications at this locus in human and rat genomes after search of human and rat segmental duplication databases. Fluorescent in situ hybridization (FISH) using a BAC containing mouse EST for *SAMD9L *(*Estm*25) revealed a single strong signal at 6A1-A2 in mouse chromosome 6 [28]. Since the sequence of *Estm*25 maps to the open reading frame of mouse *SAMD9L*, and it has very high homology with the sequence at the open reading frame of human *SAMD9*, the FISH result indicates *SAMD9 *does not exist in mouse.

Here we propose a model, which is mouse specific, for the genomic rearrangement, break in synteny, centromere formation and deletion of *SAMD9 *although it is very difficult to properly estimate all those events. We highlight a mouse specific rearrangement, resulting in the loss of the *SAMD9 *gene. Segmental duplications have been shown to mediate chromosomal rearrangements via non-allelic homologous recombination [30], and it has been shown that there is a significant correlation between low copy repeats and break of synteny between human and mouse [31]. Taken together, the existence of the mouse specific rearrangement and mouse specific low copy repeats, it would suggest a causative role, but no direct conclusions can be made. It has also been shown that centromeric regions act as reservoirs for recently duplicated sequence and this may account for the enrichment of low copy repeats at this locus [32]. As shown in Figure 7, in the mouse genome, two segmental duplications, one centromeric to *cdk6 *and the other centromeric to *SAMD9L*, may mediate chromosomal breakage with subsequent deletion of *SAMD9*, and the centromeres of mouse chromosome 6 and 5 are formed at the sites of breakage. There is no evidence of the segmental duplications at this locus in the rat genome after searching the Evan Eichler's rat segmental duplication database [33].

## Conclusion

*SAMD9 *and *SAMD9L *are expressed ubiquitously in human tissues, but they are expression at lower levels in neoplasia. *SAMD9 *regulates cell proliferation and apoptosis, and decreases tumor growth of colon cancer cell line in immune deficient mice. This is the first report as far as we know about a human gene that exists in rat, but is lost in mouse, due to a mouse specific rearrangement, resulting in the loss of the *SAMD9 *gene. Although these novel genes were identify in the study of a relatively rare disorder, aggressive fibromatosis, these genes seem to play a role regulating cell growth in a variety of tumor types.

## Methods

### Cell lines, human and mouse materials

SW480, MRC-5, Cos-1 and NIH3T3 cell lines were obtained from the American Tissue and Cell Collection. For each experiment in which cell lines were used for a quantitative assay, the experiment was conducted nine independent times, each time on a different day. The RNA populations used for subtractive hybridization were prepared from cells derived from an aggressive fibromatosis tumor with an *APC *mutation after transfection of wild-type *APC *or a control vector as previously reported [11]. Cells, tumor and normal control tissues were collected from patients with sporadic aggressive fibromatosis, colon cancer, and breast cancer while the patients were under surgical excision of the tumors. All of the samples were cryopreserved as soon as possible after resection and stored in liquid nitrogen vapor for later nucleic acids extraction. The local ethical approvals for this research were obtained.

### Suppression subtractive hybridization

Suppression subtractive hybridization was used to compare the gene expression differences in a cell culture derived from an aggressive fibromatosis tumor with an *APC *mutation after transfection of a wild type *APC *to the same cell culture after transfection of a control vector using Clontech PCR-select cDNA subtraction kit (K1804-1, Clontech, CA, USA). Both forward (cDNA from cells transfected with control vector was subtracted by cDNA from cells transfected with wild-type APC) and reverse (cells transfected with wild-type APC was subtracted by cells transfected with control vector) subtractions were conducted. There were eight genes identified in the forward subtraction and 16 genes in the reverse subtraction that were found to be differentially expressed, based on the screening of dot blots containing 196 clones in the forward and reverse subtracted libraries, respectively. *SAMD9 *was confirmed as being differentially expressed in the reverse subtraction and selected for further analysis in this paper.

### Northern analysis

15 μg of total RNA from 8 weeks, 12 weeks and full term human placenta were electrophoresed on 1% RNase-free agarose gel and Northern transferred to a nylon membrane according to according to the method from Sambrook *et al *[34]. A probe for *SAMD9 *was generated using PCR with 89729F and 88001R as primers for a 1729 bp fragment from the 3'UTR. The Northern membrane was first probed with the *SAMD9*, then stripped and probed with the human actin probe as a loading control. The probes were labeled with isotope ^32^P.

### Cloning of the full-length gene

#### (1) 5'- and 3'- rapid amplification of cDNA ends (RACE)

RACE was performed using SMART™ RACE cDNA synthesis kit (Clontech Inc., USA) according to the manufacture's instruction. The anti-sense (93769R, see Additional file 1) and the sense (93961F, see Additional file 1) primers for the 5'- and 3'- RACE were designed using the sequence of the 200 bp clone of *SAMD9 *isolated from suppression subtractive hybridization. The amplified transcripts were cloned into a pCR 2.1 vector (Invitrogen, USA), sequenced, and mapped to the DNA sequence in the GenBank.

#### (2) Long-distance PCR, cloning and sequencing

Sequences corresponding to the 5'- and the 3'- ends of the *SAMD9 *gene (106430F' and 87926R', see Additional file 1) or *SAMD9L *gene (136752F' and 119170R', see Additional file 1) were used as primers for long-distance PCR. The amplified transcripts were cloned into a pCR vector (Invitrogen, USA), and the insertion of the transcript was verified by restriction enzyme digestion and sequencing.

### PCR with human multiple tissue cDNA panels

Expression of *SAMD9 *and its paralogous gene *SAMD9L *were investigated using PCR of human multiple tissue cDNA panels including adult, fetal and tumor tissues (Clontech, USA). The sequences at the 3' UTR of the *SAMD9 *and *SAMD9L *genes were used to design the target primers according to the instruction from the manufacture since the DNase pretreated RNA was used for synthesis of those cDNA panels. 88347F and 88001R [see Additional file 1] were primers for *SAMD9*, while 119622F and 119170R [see Additional file 1] for *SAMD9L*. *GAPDH *was used as a control.

### Semi-quantitative RT-PCR

The total RNA was extracted from tumor tissue and normal control tissue from aggressive fibromatosis, breast and colon cancers using Trizol RNA reagents (Invitrogen, USA). The anti-sense and sense primers for *SAMD9 *and human beta-2 microglobulin (*beta 2M*) were designed using the sequence across the introns, and *beta 2M *was used as control. For *beta 2M*, beta 2M-F was used as sense primer and beta 2M-R as anti-sense primer to produce a 112 bp PCR product [see Additional file 1]. For *SAMD9*, 103270F was used as sense primer and 93871R as anti-sense primer to produce a 711 bp PCR product for *SAMD9 *with SAM domain coding sequence and a 222 bp PCR product for *SAMD9 *lacking SAM domain coding sequence [see Additional file 1]. The optimal amplification cycles were set within the linear range, 30 cycles for *beta 2M *and 37 cycles for *SAMD9*, after applying series of diluting cDNA samples and subjecting for series of PCR amplification cycles.

The polymerase cycling reactions of paired cDNA samples were conducted at the same time for both target and control genes. The amplified products were electropheresed on a 2% agarose gel and photographed under UV light. The expression of *beta 2M *was set as control to normalize the level of target expression. The expression level of the target gene *SAMD9 *was analyzed using computer software ImageQuant and represented as the ratio of its intensity verse the intensity of the *beta 2M *gene under the condition that the same amount of the cDNA was applied as template for the target gene and the control gene.

### Real-time quantitative PCR

Real-time quantitative PCR was undertaken using 28S rRNA (*28S*) as the control gene. PCR primer pairs for human 28S rRNA were taken from Simpson *et al*, 2000 [35], for *SAMD9*, 103270F was used as sense primer and 94424R as anti-sense primer, and for *SAMD9L*, 133857F was used as sense primer and 124170R as anti-sense primer [see Additional file 1]. Validation curves were carried out for the primer sets using RNA from SW480 cells diluted to 1:5, 1:10, 1:50, 1:100, and 1:1000. Delta delta Ct method was used for setting up the experiment and analysis of the data. An arbitrarily designed threshold was set at 0.2 for all analysis, while the baseline cycles were set for all analysis from 3 to 10 cycles for *28S *and from 3 to 30 cycles for *SAMD9 *or *SAMD9L*. The threshold cycle, C_t _was determined using the analysis software (SDS 2.1, Applied Biosystems). The result was analyzed using the relative quantitative 2^-ΔΔ C ^_t _method [36]. The expression level of the target gene *SAMD9 *or *SAMD9L *in tumor tissues were represented as the fold difference from normal control tissues.

### Generation of expression vectors

#### (1) Long distance PCR amplification of SAMD9 or SAMD9L open reading frame

The sequences flanking the predicted open reading frame of the *SAMD9 *or *SAMD9L *gene were used as primers. For *SAMD9*, 94504F was the sense primer and 89735R was the anti-sense primer, while 124378F as sense primer and 119624R as anti-sense primer for *SAMD9L *[see Additional file 1]. The predicted open reading frame of the *SAMD9 *or *SAMD9L *was subcloned into pDNR-1r donor vector using standard methods [34]. The correct orientation of the insert was confirmed by restriction enzyme digestion and sequencing to generate pDNR-1r-SAMD9-ORF, pDNR-1r-SAMD9L-ORF-major and pDNR-1r-SAMD9L-ORF-minor.

The pDNR-1r-SAMD9-ORF, pDNR-1r-SAMD9L-ORF-major and pDNR-1r-SAMD9L-ORF-minor were subcloned into several acceptor vectors, such as pLP-EGFP-C1 and pLP-CMV-HA (Clontech, USA), using Clontech Creator™ DNA cloning kit (PT3460-2, Clontech, USA) with Cre recombinase through Cre-loxP site-specific recombination to catalyze the transfer of the open reading frame from pDNR-1r donor vector to the acceptor vectors according to the manufacture's instruction.

### Western blot analysis with anti-GFP

The total protein from Cos-1 cells transfected with either pLP-EGFP-SAMD9 or its control vector pLP-EGFP were harvested using Nonidet P-40 lysis buffer (1% Nonidet P-40, 50 mM Tris-HCL [pH 7.5], 150 mM NaCl, 2 mM EDTA, 10% glycerol, proteinase inhibitor tablet [1 tablet per 10 mL buffer, Roche, USA]). The protein was electropheresed on 8% sodium dodecyl sulphacrylamide gel and transferred to membrane according to the method from Sambrook et al, 1989. The membrane was then probed with anti-GFP (1:200, Santa Cruz, USA) and secondary antibody (HRP-conjugated monoclonal anti-mouse IgG, 1:2000, Transduction Laboratory, USA). Finally, the membrane was exposed to X-ray film for 20 minutes. The total protein from Cos-1 cells transiently co-transfected with pLP-EGFP-SAMD9 and either one of the two *SAMD9 *RNA interference vectors (pSUPER-RNAi-SAMD9, pSUPER-RNAi-SAMD9-2^nd^), RNA interference vector with mutated *SAMD9 *sequence (pSUPER-RNAi-SAMD9-mut9) to null its function as negative control, or its control empty vector (pSUPER-RNAi-EGFP) were also harvested for Western blot analysis to confirm the knock-down of *SAMD9 *expression by RNA interference.

### Generation of RNA inference vector for SAMD9

After searching of the public available sequence database, a 19 bp oligonucleotide sequence (846 bp after the start of translation) specific for the RNA inference of the *SAMD9 *expression was found (5'-GTGCATTCGAGAGCCAAGA-3') and subcloned into a pSUPER-RNAi-EGFP expression vector to generate the *SAMD9 *RNA interference vector (pSUPER-RNAi-SAMD9). A nucleotide substitution from G to A at the 9^th ^position of this 19 bp oligonucleotide results in abolishing of its specificity to *SAMD9 *to generate the negative control vector (pSUPER-RNAi-SAMD9-mut9). Another 19 bp oligonucleotide sequence (2371 bp after the start of translation) specific for the RNA inference of the *SAMD9 *expression was found (5'-TACGTACCTGTACTACTCC-3') and also subcloned into a pSUPER-RNAi-EGFP expression vector to generate a second *SAMD9 *RNA interference vector (pSUPER-RNAi-SAMD9-2^nd^).

### Cell culture, gene transfection, selection, flow cytometry cell sorting, immunoflurescent staining and confocal microscopy

SW480, MRC-5, or Cos-1 cells (ATCC, USA) were cultured in the DMEM medium with 10% fetal calf serum (Invitrogen, USA) in a CO_2 _incubator at 37°C. Gene transfection was conducted using Fugene 6 reagent (Roche, USA). The transfected cells were either harvested for transient transfection or selected with the addition of 200 ng of G418 (Sigma, USA) for 2 weeks, and sorted by flow cytometry using GFP as marker. The cellular location of *SAMD9 *was analyzed under a confocal microscope in Cos-1, MRC-5 and SW480 cells transfected with pLP-EGFP-SAMD9. The effectiveness of *SAMD9 *RNA interference was documented by using confocal microscopy in SW480 cells co-transfected with pLP-CMV-HA-SAMD9 and either the *SAMD9 *RNA interference vector (pSUPER-RNAi-SAMD9) or its empty control vector (pSUPER-RNAi-EGFP), which were fixed, stained with anti-HA (1:200, Santa Cruz, USA) and Tex-red conjugated secondary antibody (1:100, Molecular Probes, USA).

### *In vitro *assays

#### (1) Cell proliferation assay

Cells were seeded at the 2.5 × 10^4 ^cells/mL on glass cover slips and cultured in DMEM with 10% fetal bovine serum at 37°C. S-phase cells were labelled overnight by supplementation of the cell culture medium with BrdU (Sigma, St louis, MO; 10 μM) and detected by mouse monoclonal anti-BrdU (DAKO, Demark; 1:100) as the published method [37]. After the final step, the glass cover slips were removed from the well and mounted on microscope slides by mounting medium with DAPI to stain the nuclei. The number of S-phase cells and total number of nuclei were counted over five fields at center and four corners on the glass cover slip at 100× magnification. Each experiment was conducted nine times.

#### (2) Cell motility and invasion assays

Cells were seeded at 5 × 10^4 ^cells/mL and cultured with DMEM and 10% fetal bovine serum for 22 hours at 37°C in the BioBoat^® ^culture inserts (BD Bioscience) for motility assay and BioBoat^® ^Matrigel invasion chambers (BD Bioscience) for invasion assay. After the incubation period, cells on the upper surface of the membrane were removed, but cells that migrated to the lower surface of the membrane were fixed to the membrane with 4% paraformaldehyde. The membranes were then excised from the insert and mounted on microscope slides by mounting medium with DAPI to stain the nuclei. The total number of nuclei was counted in each of five fields across the diameter of the member viewed at 400× magnification using UV fluorescence microscopy. The experiments were conducted nine times.

#### (3) Cell apoptosis assay

Cell apoptosis activity was studied using caspase-3 colorimetric activity assay kit (Chemicon International, USA). The experiments were conducted nine times and relative caspase-3 activity was represented as percentage changes of the treatment group to the control group and calculated by dividing the optical density reading of the treatment group to the control group and multiplying 100.

### Xenograft in immune deficient mice

#### (1) SW480

The flow cytometry sorted SW480 cells transfected with either pLP-EGFP-SAMD9 or its control vector pLP-EGFP were suspended in 1× PBS (120 mM NaCl, 10 mM Na_2_HPO4, 3 mM KH_2_PO_4_, pH 7.3) and injected underneath the skin on the back of nude mice and NOD-SCID mice at a concentration of 2.5 million cells per injection. A similar explant was performed using sorted SW480 cells co-transfected with pLP-CMV-HA-SAMD9 and the *SAMD9 *RNA interference vector (pSUPER-RNAi-SAMD9) or pLP-CMV-HA-SAMD9 and the empty control vector (pSUPER-RNAi-EGFP). The mice were sacrificed after 1 week of injection and the size of tumors were blindly scored.

#### (2) MRC-5

The flow cytometry sorted MRC-5 cells transfected with the *SAMD9 *RNA interference vector (pSUPER-RNAi-SAMD9) or the empty control vector (pSUPER-RNAi-EGFP) were also suspended in 1× PBS and injected underneath the skin on the back of NOD-SCID mice at a concerntration of 5.9 million cells per injection. The mice were sacrificed after 1 week of injection and the size of tumors were blindly scored.

### Statistical analysis

The counting process was conducted by a blinded observer. Means and 95% confidence intervals were calculated. The t-test was applied to compare differences.

## New sequence accession numbers

GenBank: AF445355, AF474973, AF453311, AY195582-195587, DQ068177.

## Authors' contributions

CFL carried out the subtraction hybridization, the initial experiments cloning the genes, generating the expression and siRNA constructs, and drafting the manuscript, JRM carried out the analysis of the genes in different species, and assisted in drafting the manuscript. RYW carried out experiments to determine the expression levels of *SAMD9 *and *SAMD9L *in normal and tumor tissues, JR carried out experiments to determine the *SAMD9L *splice variants, KL carried out gene transfection experiments, CK carried out cell proliferation experiments, RK carried out experiments to detect *SAMD9 *using Western analysis, SB carried out experiments to detect *SAMD9 *using Northern analysis, SWS carried out the analysis of the localization on chromosome seven, participated in the design of the study, and assisted with the draft of the manuscript. BAA conceived of the study, and participated in its design and coordination and helped to draft the manuscript. All authors read and approved the final manuscript.

## Supplementary Material

Additional file 1 Supplementary Results and a summary of Primers used in the cloning of these genes. The data provided represents additional details on the structure and splice variants of SAMD9 and SAMD9L. In addition, there is a table listing the PCR primers used in this work. Click here for file

## Figures and Tables

**Table 1:** Summary of orthologous genes of *SAMD9 *and *SAMD9L *in multiple species

***SAMD9***		***SAMD9L***	
Species	Accession	Species	Accession

Human	GenBank: NP060124.2	Human	GenBank:NP_689916.1
Chimpanzee	aligns to genome^b^	Chimpanzee	GenBank:XP_527818.1
Dog	aligns to genome^b^	Dog	GenBank:XP_539422.1
Rat	GenBank:XP_575365.1	Rat	GenBank:XP_575369.1
Mouse	none^a^	Mouse	GenBank:XP_620286.1
Chicken	GenBank:XP_418660.1	Chicken	none^a^
Cow	GenBank:XP_592017.1	Cow	none^a^
Zebrafish	GenBank:XP_698198.1	Zebrafish	none^a^
Xenopus tropicalis	GenBank:AAH91702	Xenopus tropicalis	none^a^
Xenopus laevis	GenBank:AAH94169	Xenopus laevis	none^a^
Tetraodon nigroviridis	GenBank:CAF92907	Tetraodon nigroviridis	none^a^

^a ^There is no match in protein or nucleotide database. ^b ^There is best reciprocal nucleotide match found in genomic sequence.
