# Supplementary material for: Human sterile alpha motif domain 9, a novel gene identified as down-regulated in aggressive fibromatosis, is absent in the mouse
Source: BMC Genomics. 2007 Apr 3;8:92. doi: 10.1186/1471-2164-8-92 (PMC1855325; doi:10.1186/1471-2164-8-92)
Supplement: Additional file 1 — Supplementary Results and a summary of Primers used in the cloning of these genes. The data provided represents additional details on the structure and splice variants of SAMD9 and SAMD9L. In addition, there is a table listing the PCR primers used in this work. [file 1471-2164-8-92-S1.pdf]

Additional file 1

Title: Supplementary Results and a summary of Primers used in the cloning of these genes

Splice variation of SAMD9

In addition to the transcript described in the main text, a transcript variant [GenBank: AF453311] was isolated from 5'- RACE of the *SAMD9* gene, 5'RACE1-1 (Supplementary Fig. 1), in which the transcriptional initiation site starts later than the above full-length transcript and the third exon is spliced into exon 3A and 3B. Due to this alternative splicing in the coding region for SAM domain, the predicted putative protein does not have a SAM domain. However, the full-length transcript of this variant has not been cloned. Using the sequence of this transcript variant compared to the genomic sequence, the composition of exons is 125 bp, 101 bp, and 90 bp for exon 1, 2 and 3A, respectively, and the sequence following exon 3A stays as intron 3, which contains 489 bp (Supplementary Fig. 3A-B). The non-canonical dinucleotides AT and TC are donor and acceptor splice sites for intron 3, and there is an in-frame canonical pair of the donor and acceptor (GT and AG) splice sites near by (Supplementary Fig. 3C).

RT-PCR of 42 normal tissues and 37 tumor tissues were conducted with primers across the second and third introns. Although there was no amplification in 11 samples including 5 normal and 6 tumor tissues, the large fragment corresponding to the size of the transcript annotated with a SAM domain was found in 66 of 68 amplified samples (97%), while the small fragment corresponding to the size annotated without a SAM domain was detected in only 18 of 68 amplified samples (26%). All of the fragments were isolated and sequenced. The sequencing

results showed that they correspond to two transcripts of *SAMD9*, one with and the other without the putative SAM domain. This suggests that the transcript with the SAM domain is the primary transcript.

Splice variants of SAMD9L

There were ESTs and experimental evidence to support the alternative transcript variants of 5' exons comprising the un-translated region (UTR) of *SAMD9L*. After performing RT-PCR of *SAMD9L* using the 5'- end sequence as sense primer and the sequence 208 bp after the translation start site of the *SAMD9L* gene as anti-sense primer, 136750F and 124170R, seven different fragments were found in the SW480 cells (Supplementary Fig. 4A-B). All fragments were isolated and sequenced (Supplementary table 1). The sequencing results showed that there were six splice variants of *SAMD9L* [GenBank: AY195582-195586, DQ068177]. These results also show that one of the transcripts, 822 bp with six exons (152 bp, 59 bp, 132 bp, 148 bp, 102 bp and 229 bp) and five introns (1047 bp, 1047 bp, 376 bp, 551 bp and 8740 bp), matches the above full-length sequence of *SAMD9L* [GenBank: AF474973].

As shown in supplementary table 1, *SAMD9L* splice variant 1 [GenBank: AY195583] has 381 bp with two exons (152 bp for exon 1 and 229 bp for exon 6) and one intron (12202 bp); splice variant 2 [GenBank: AY195582] has 631 bp with four exons (152 bp, 148 bp, 102 bp and 229 bp for exon 1, 4, 5 and 6) and three introns (2661 bp, 551 bp, and 8740 bp); splice variant 3 [GenBank: AY195584] has 1027 bp with six exons (152 bp, 264 bp, 132 bp, 148 bp, 102 bp and 229 bp for exon 1, 2A, 3, 4, 5 and 6) and five introns (1047 bp, 842 bp, 376 bp, 551 bp and 8740 bp); splice variant 4 [GenBank: AY195585] has 1198 bp with five exons (152 bp, 59 bp, 656 bp,

102 bp and 229 bp for exon 1, 2, 3A, 5 and 6) and four introns (1047 bp, 1047 bp, 551 bp and 8740 bp); splice variant 5 [GenBank: AY195586] has 1403 bp with five exons (152 bp, 264 bp, 656 bp, 102 bp and 229 bp for exon 1, 2A, 3A, 5 and 6) and four introns (1047 bp, 842 bp, 551 bp and 8740 bp); splice variant 6 [GenBank: DQ068177] has 763 bp with five exons (152 bp, 132 bp, 148 bp, 102 bp and 229 bp for exon 1, 3, 4, 5 and 6) and four introns (2153 bp, 376 bp, 551 bp and 8740 bp) (Supplementary Fig. 4C, 5). All seven different transcripts of *SAMD9L* were amplified in SW480 cells and only splice variant 5 and 6 were found in normal colon mucosa. Exon 2A has the same starting sequence as exon 2, but ending later than exon 2 because 205 bp of the intron sequence following exon 2 is also transcribed in exon 2A. Exon 3A has the same starting sequence as exon 3 and same ending sequence as exon 4 because 376 bp of intron 3 sequence is transcribed in exon 3A. Exon 3A contains the sequence for exon 3, intron 3 and exon 4, altogether.

A 771 bp transcript variant of *SAMD9L* [GenBank: AY195587] was also amplified from aggressive fibromatosis tumor tissue using the sequences from the most 5’- and 3’- EST in the region. However, the coding region of this transcript variant is not determined. Using the sequence of this transcript compared to the genomic DNA sequence from GenBank, there are three exons (152 bp, 169 bp, and 450 bp for exon 1, 2B and 6B) and two introns (1047 bp and 15765 bp) (Supplementary table 1, Supplementary Fig. 5). Exon 2B has the same start sequence as exon 2, but ending later than exon 2 because 110 bp of the intron sequence following exon 2 is also transcribed in exon 2B. Exon 6B starts later and ends early than exon 6.

Supplementary figure legends

Supplementary figure 1. Sequences of EST and RACE mapped to the region for *SAMD9*. Sequences of EST and RACE mapped to the region for *SAMD9*. The sequences of EST and RACE clones were mapped to the sequence of a BAC clone [GenBank: AC000119] shown as black box, which codes the genomic sequence of *SAMD9*. The most 5'- and 3'- ends of sequence for *SAMD9* was found in 5'RACE clone 1-6 and an EST clone [GenBank: AA628487], respectively. The transcript variant of *SAMD9* lacking coding sequence for SAM domain was found in 5'RACE clone 1-1 shown as four light blue lines, representing 4 exons. The *SAMD9* clone (AF5-12) identified in suppression subtractive hybridization is shown as red line. The 5'-RACE clones are shown as blue lines and 3'- RACE clones are shown as pink lines. The EST clones from GenBank are shown as green lines. The EST clones from Celera database are shown as yellow lines. The sequenced EST clone [GenBank: AI370412] is shown as black line. The orange line represents the initial clone identified by subtraction hybridization.

Supplementary figure 2. Sequences of EST mapped to the region for *SAMD9L*. The sequences of EST clones were mapped to the sequence of a BAC clone [GenBank: AC000119] shown as black box, which codes the genomic sequence of *SAMD9L*. The most 5'- and 3'- ends of sequence for *SAMD9L* were found in the EST clones [Celera Database: THC511215 and THC513290, respectively]. The EST clones from GenBank and Celera database are shown as lines.

Supplementary figure 3. Transcript splicing variant of *SAMD9*. A. Diagram of the *SAMD9* transcript splicing variant. Exons are shown as red boxes. **B.** RT-PCR of normal and tumor tissues were conducted with primers across the second and third introns. Alternatively spliced transcript was seen in one of the normal fibrous tissues. **C.** Diagram of the sequences at the exon 3A - intron 3 - exon 3B junctions of *SAMD9* gene. The non-canonical **AT-TC** pair was found as donor and acceptor sites in the intron 3 for the alternatively splicing of *SAMD9*. An in-frame canonical **GT-AG** pair is close by. The red rectangles represent exons.

Supplementary figure 4. Transcript splicing variants of *SAMD9L*. A. Diagram of *SAMD9L* transcript splicing variants. Exon was shown as red box. **B.** *SAMD9L* transcript splicing variants. After performing RT-PCR of *SAMD9L* using the 5'- end sequence as sense primer (136750F) and the sequence 208 bp after the start of the open reading frame of the *SAMD9L* gene as anti-sense primer (124170R), seven different fragments were found in SW480 cells.

Supplementary figure 5. Diagram of *SAMD9* and *SAMD9L* transcripts in human, mouse and rat. Exons are shown as black boxes. There is no transcript or EST in mouse for *SAMD9*.

Supplementary figure 6. Phylogram of orthologous genes of *SAMD9* and *SAMD9L* in multiple species. Evidence for *DRIF-1* (*SAMD9*) was found for (chichen/frog/fish), while *DRIF-2* (*SAMD9L*) is currently only found in mammalian species. No evidence for either gene was

found in lower eukaryotes such as Drosophila, C. elegans or yeast. This would suggest that *SAMD9* is the ancestral gene, which has been subsequently lost in the murine lineage.

Supplementary figure 7. Mouse segmental duplication in the region of *SAMD9L*. There are segmental duplications flanking a mouse EST clone [GenBank: AA175286].

Supplementary figure 8. Phylogram of human *SAMD9*, human *SAMD9L* and a mouse EST clone. The mouse EST clone [GenBank: AA175286] is mouse EST for *SAMD9L*.

Supplementary table 1. Summary of *SAMD9L* alternative splicing transcripts

Transcript name	GenBank accession number	Exon 1(bp)	Exon 2(bp)	Exon 3(bp)	Exon 4(bp)	Exon 5(bp)	Exon 6(bp)
SAMD9L	GenBank: AF474973	152	59	132	148	102	5228
SAMD9L splice variant 1	GenBank: AY195583	152					229
SAMD9L splice variant 2	GenBank: AY195582	152			148	102	229
SAMD9L splice variant 3	GenBank: AY195584	152	264 (exon 2A)	132	148	102	229
SAMD9L splice variant 4	GenBank: AY195585	152	59	656 (exon 3A)		102	229
SAMD9L splice variant 5	GenBank: AY195586	152	264 (exon 2A)	656 (exon 3A)		102	229
SAMD9L splice variant 6	GenBank: DQ068177	152		132	148	102	229
transcript variant of *SAMD9L*	GenBank: AY195587	152	169 (exon 2B)				450 (exon 6B)

Supplementary table 2. Summary of Primer Sequences

Primer	Sequence
103270F	5’-GTG GCC TTT TGT GAT CTC CT-3’
106430F’	5’-GAC AGA AGC AGA GAA TAC ATA-3’
119170R’	5’-TAT TGA TAC CAA GAT GAG GTA A-3’
119622F	5’-ACA ATA CAT CAC CTG TAG TTC-3’
119624R	5’-TTA AAT TAC TTC TAT ATC ATA TGC-3’
124170R	5’-GAC TAA TCC TGT TAC TTC TTC-3’
124378F	5’-ATG AGT AAA CAA GTA TCT CTA-3’
133857F	5’-AAC AGC CTC ATG AAG ACT TAT-3’
136752F’	5’-AGA AGA CTT CAC AAT TTA ATG C-3’
28S-F	5’-TTG AAA ATC CGG GGG AGA G
28S-R	5’-ACA TTG TTC CAA CAT GCC AG
87926R’	5’-AAT ATA CAA TTT TTA TTT GTC AAT TA-3’
88001R	5’-TGC AGA AGC AAG GGA AAA TC-3’
88347F	5’-ATT GAA GAT TCC ATG GTT ATG T-3’
89729F	5’-TGA TAT TCT TCC TCC AAG AAT T-3’
89735R	5’-TTA AAC AAT TTC AAT GTC ATA AGC-3’
93769R	5’-TCC CAT GGG GTT TGT CTT TGA CTC CA-3’
93871R	5’-TGA CAT CCT CTT CTG TGG CT-3’
93961F	5’-CAG CCT GAA ACA GGA CCA GGC AAT CTC A-3’
94424R	5’-CTT ATG ACT TTC TAA CCA CTG A-3’
94504F	5’-ATG GCA AAG CAA CTT AAC CTT-3’
beta 2M-F	5’-ACC CCC ACT GAA AAA GAT GA-3’
beta 2M-R	5’-ATC TTC AAA CCT CCA TGA TG-3’
m102433F	5’-TGT AGA AGA AAT TTG AAA ATT TGG-3’
m80349R	5’-TGT TCT TTG GTC CAG TCT TTG-3’

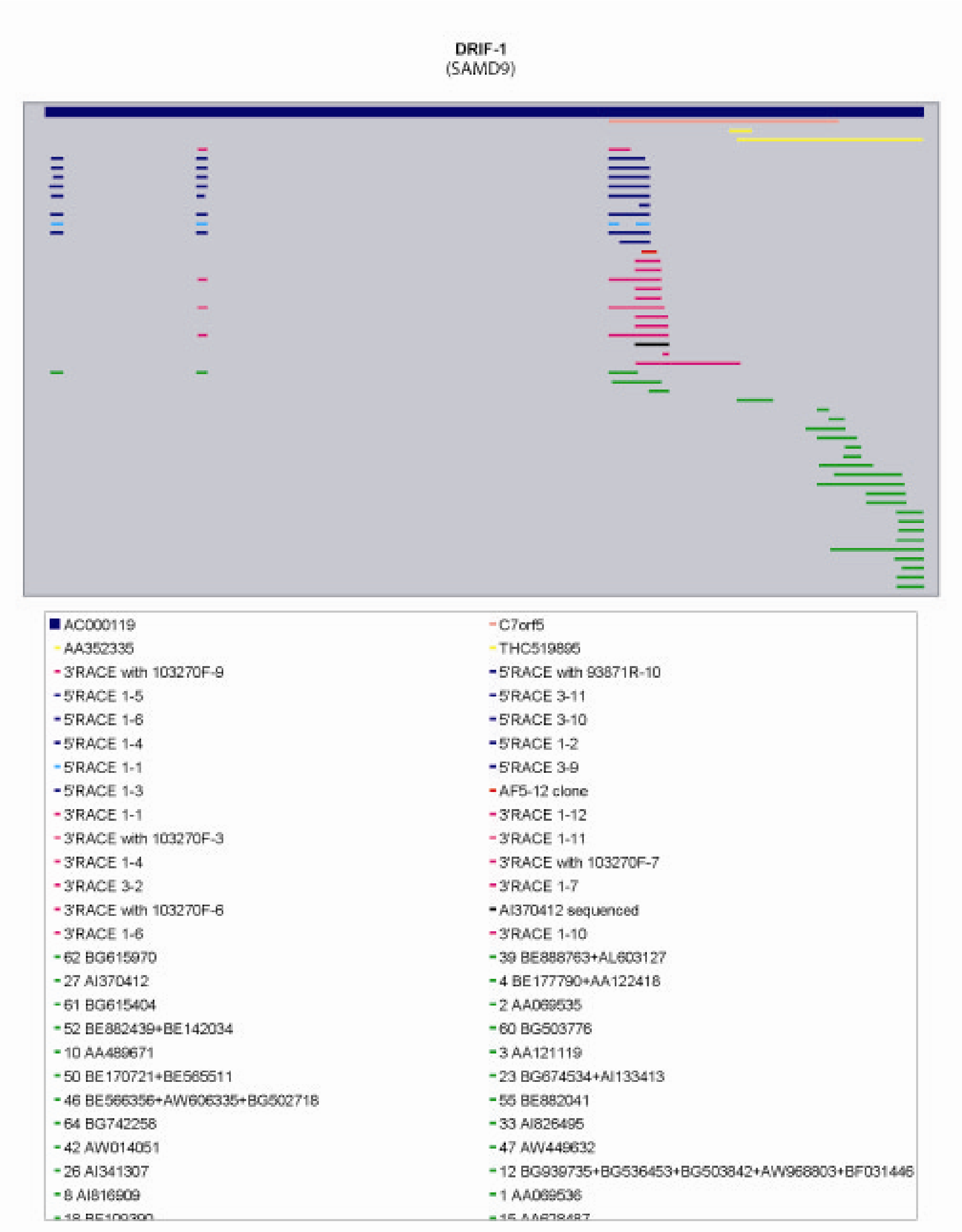

Suppl Figure One

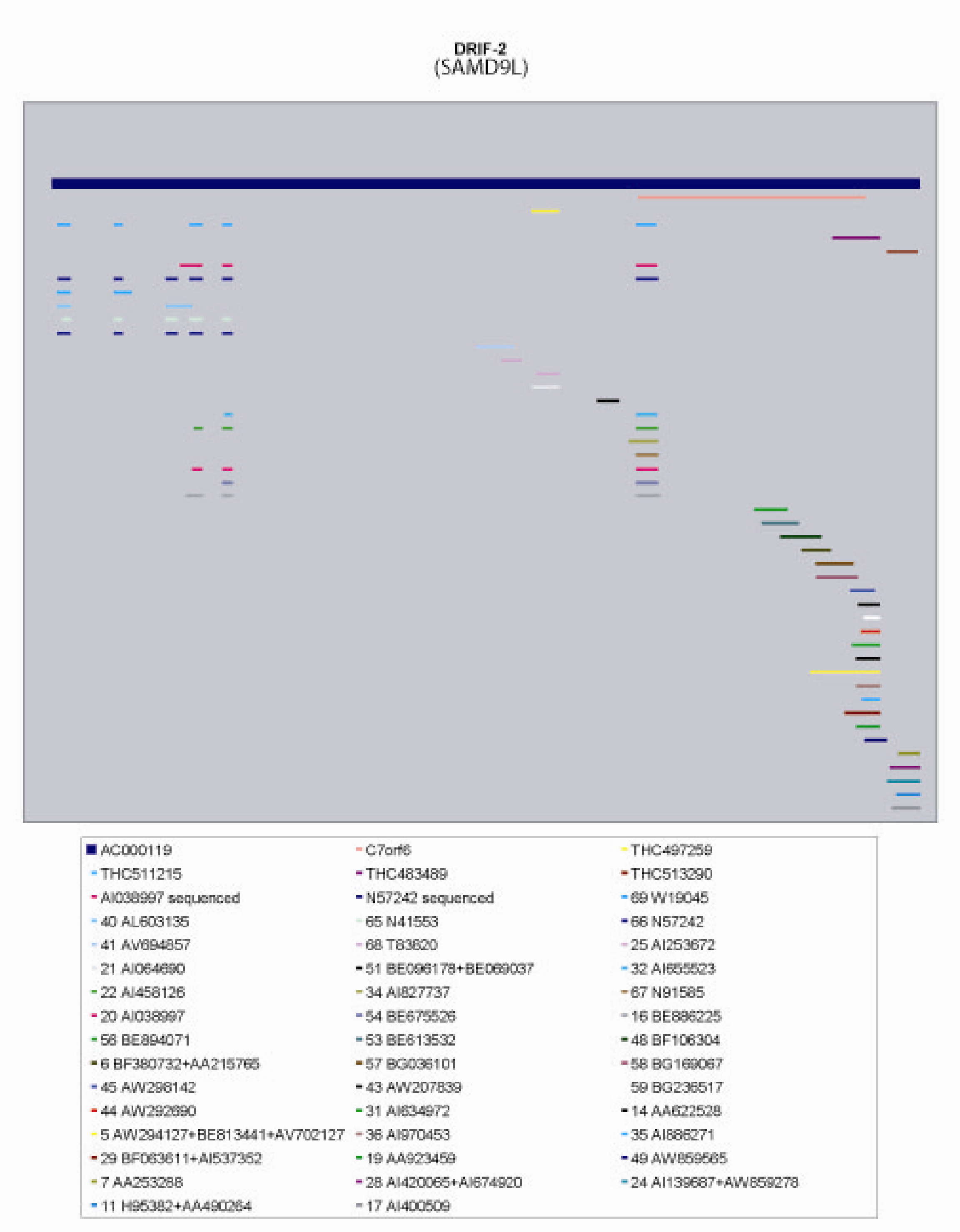

Suppl Figure two

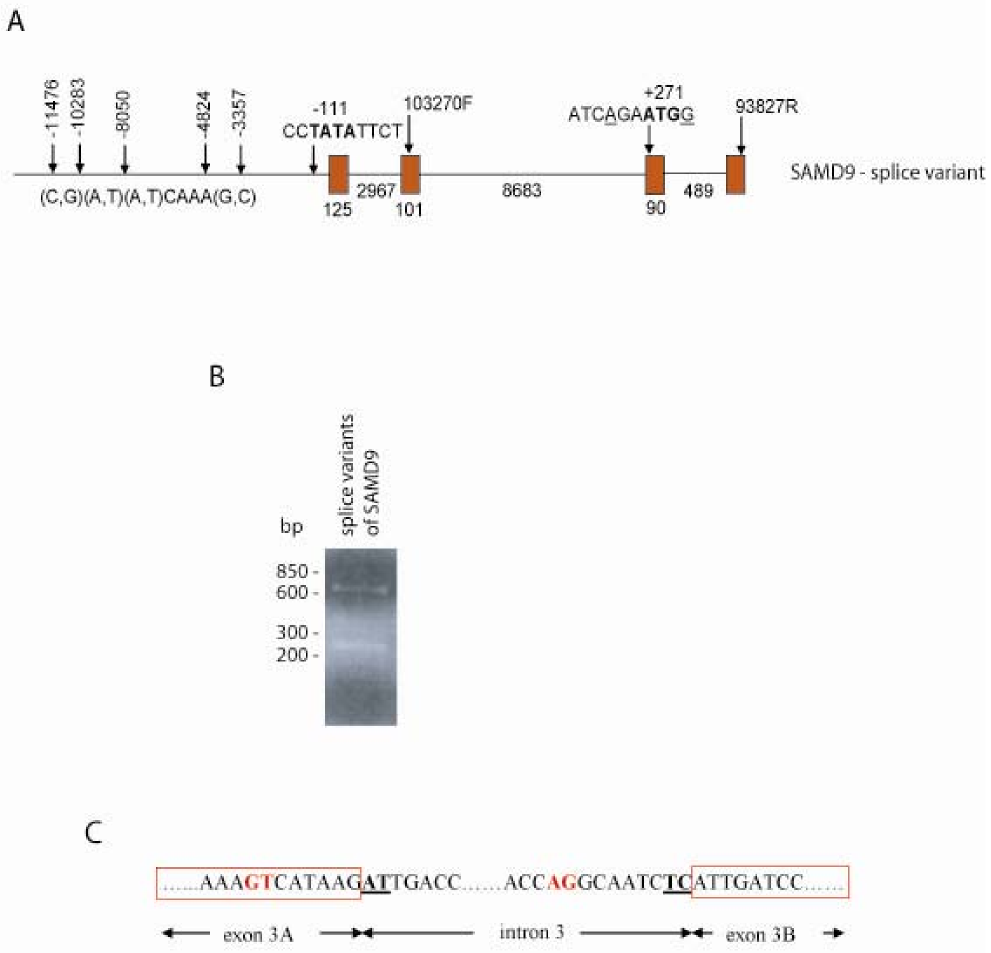

Suppl Figure Three

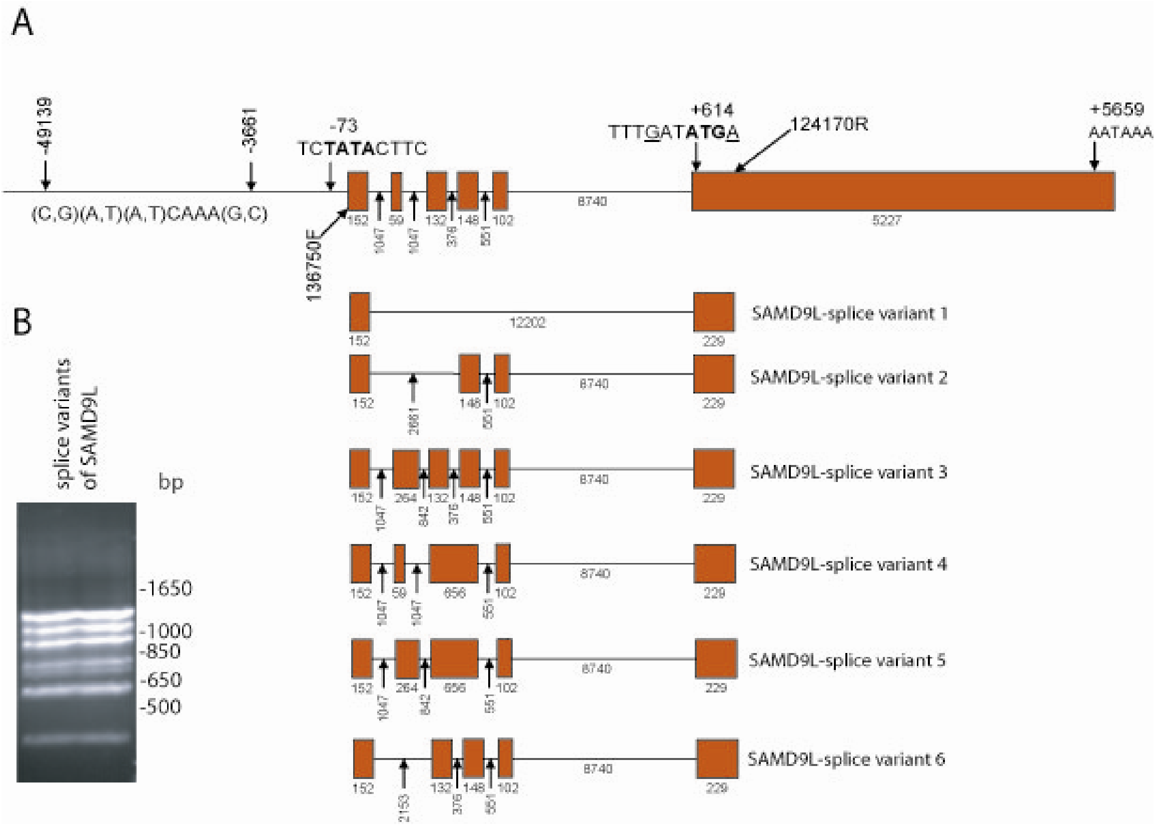

Suppl Figure Four

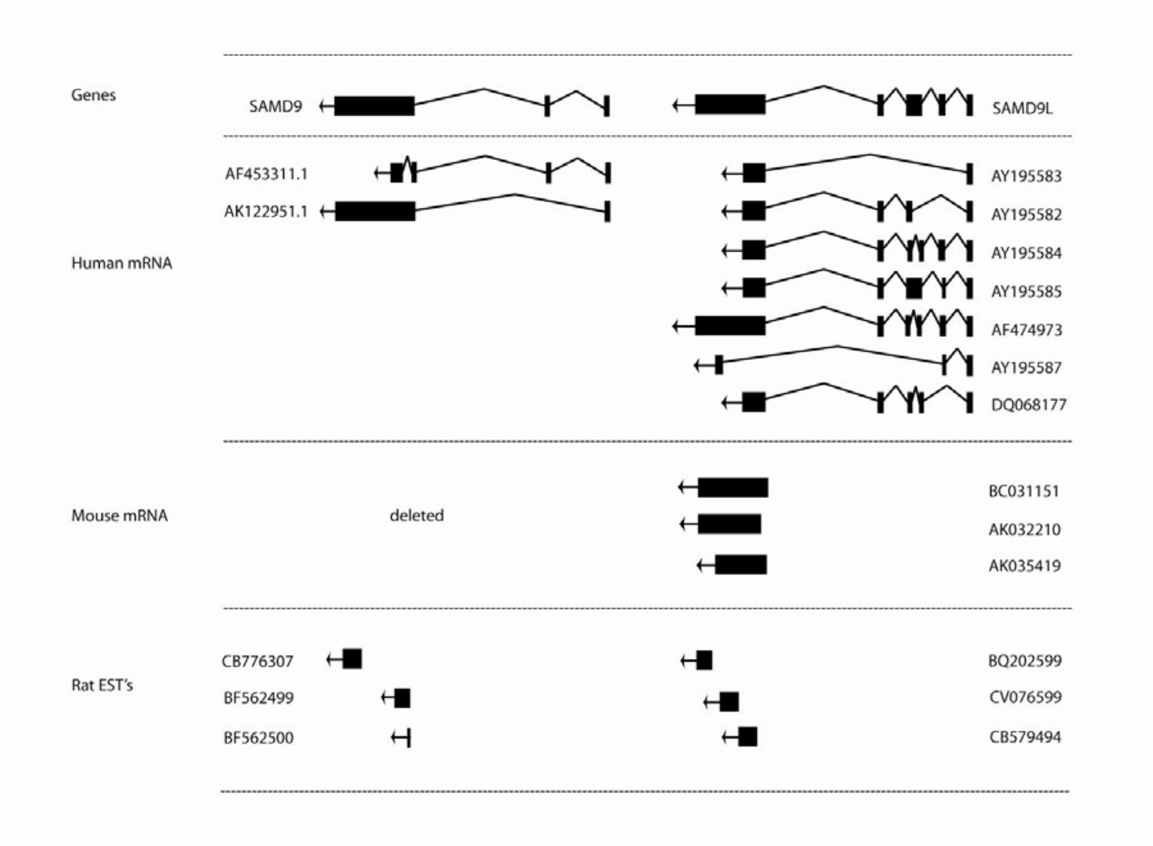

Suppl Figure Five

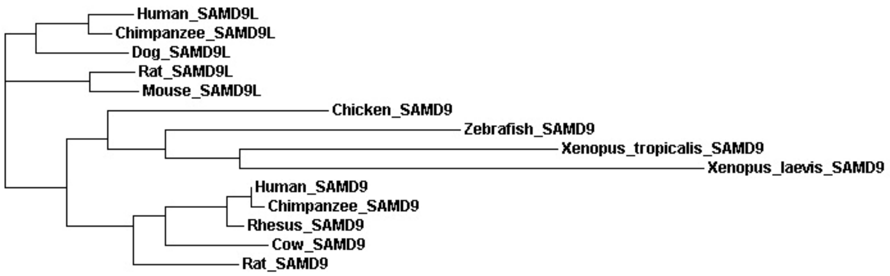

Suppl Figure Six

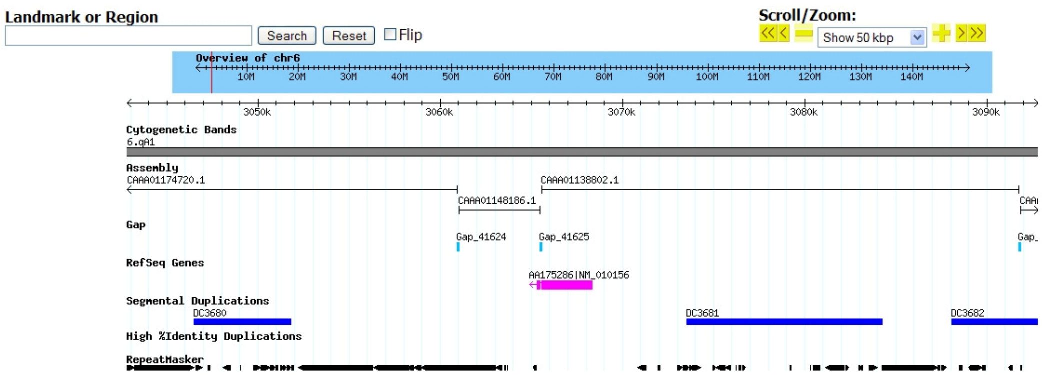

Suppl Figure Seven

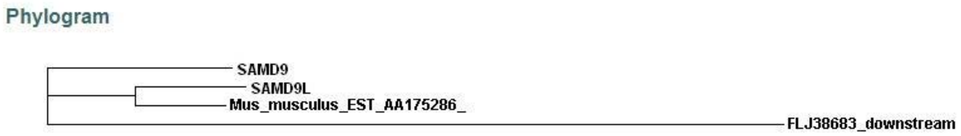

Suppl Figure Eight
